# A Picture of Trait Anxiety and Aggressiveness among Adolescents from Different Types of Educational Institutions

**DOI:** 10.11621/pir.2021.0409

**Published:** 2021-05-17

**Authors:** Sofya Yu. Tarasova

**Affiliations:** a Psychological Institute of Russian Academy of Education, Moscow, Russia

**Keywords:** Aggressiveness, hostility, trait anxiety, perfectionism, destructive tendencies of personality

## Abstract

**Background:**

This study is an attempt to identify psychological markers for pupils’ maladaptive states. Agggression is seen as behavior while aggressiveness is seen as the state of being ready for such behavior. Anxiety is considered a stable personality trait. Two hundred and sixty pupils of the 8th grade from five different schools took part in the research. Schools that have a selection principle for admission are called prestige schools.

**Objective:**

The purpose of this study was to discover correlations between trait anxiety and aggressiveness among adolescents in different types of educational institutions. At the same time, we were interested in manifestations of maladaptive perfectionism and destructive personality tendencies as revealed by quantitative and qualitative research methods.

**Design:**

The first stage of the study was a survey to define risk groups for hostility and anxiety among the adolescents. All tests except the one for sociometry were done on an individual basis. The second stage included methods for qualitative analysis of personality. The pupils from identified risk groups were paid special attention. We were interested in the recurrence of destructive personality tendencies as they appeared in each method.

**Results:**

About one third of all the participants from four out of the five schools formed the hostility risk group. In the prestige and non-prestige schools alike, hostile adolescents were more likely to find themselves at least somewhat isolated in their social group (class). Hostile teenagers have more self-esteem problems and issues with forming a Self-concept in general, as well as pronounced destructive personality tendencies in some cases. However, the pictures of trait anxiety in prestige and non-prestige schools were different. Hostile teenagers from non-prestige schools were more likely to greatly underestimate their intelligence and communication skills. In this case, according to the teachers’ expert opinion and the results of participant observation, that assessment reflected the reality.

The hostile teenagers at the prestige educational institutions presented a different picture of trait anxiety. Objectively, the achievements of these pupils were great, but they constantly procrastinated, didn’t believe in success, and had communication problems.

**Conclusion:**

This study has shown that the markers of a pupil’s maladaptive state are trait anxiety (school, interpersonal, self-esteem), hostility, and maladaptive perfectionism.

## Introduction

The number of auto-aggressive manifestations among pupils, children, and adolescents has increased over the last years, resulting in deliberate self-injury, toxic substances overdose, etc. This phenomenon can be found in different kinds of educational institutions and educational systems. Non-suicidal auto-aggression can be in the form of deliberate and accidental self-injury, dissatisfaction with one’s own body, and eating disorders. Each finalized suicide of a youth 25 years old or younger occurs after a number of auto-aggressive manifestations.

According to the Serbsky Center, suicide mortality in our country is about 1.5 times higher than murder mortality and almost two times higher than that from car accidents. The Serbsky Center reports that over the last 20 years, the frequency of suicides among young people in Russia has increased by three times, and among children and adolescents ages 5 to 14 years, by eight times (Federal State Statistics Service, 2009). Over the last 20 years finalized suicide in Russia has been recorded at more than 20 cases per 100 thousand people (Polozhy, & Vasiliev, 2014). As elsewhere in the world, the highest increase in suicide frequency has been recorded in the age group of 10 to 24 years old (Polozhy, & Vasiliev, 2014). But what are the psychological markers of such maladaptive behavior? Is their early detection possible?

Like A.M. Prikhozhan, we consider anxiety to be a stable personality trait with a pronounced adaptive nature (Prikhozhan, 2009). Children’s anxiety is associated with defending their habitual impressions about themselves, self-esteem, and Self-concept. The Psychology Dictionary edited by A.V. Petrovsky and M.G. Yaroshevsky offers the following definitions for the above-mentioned terms: “Self-esteem is the assessment of oneself, one’s potential, qualities, and place in society by a personality,” (1990, p. 352) while Self-concept is an integral multilevel system of impressions a person has about himself/herself and about what he or she is doing (*ibid*.). Of course, as a component of Self-concept, self-esteem largely depends on a person’s socio - cultural context. It is being defined and corrected continuously during communication and the usual course of life, *i.e.,* it’s dynamic.

Thanks to a sense of self-esteem, a person can balance his or her resources for their purposes and tasks; this is how its regulatory function works. The self-esteem of anxious people is unstable and full of conflict; they don’t believe in success. Moreover, anxious people often stick to a low self-concept because it allows them to avoid making uncomfortable choices: “I won’t succeed anyway. There is no point in trying. I’m a loser.” Anxiety becomes a psychological defense mechanism. At the same time, a free-floating, generalized anxiety reflects an unrealized need for stable positive self-esteem. Thus, a neurotic vicious circle appears.

In our thesis research, we defined the risk group for school anxiety and maladap-tation as follows: secure, inadequately anxious children. These children demonstrate high performance, and have good a appearance and social status, but they manifest constant super-anxiety (Tarasova, 2015). On the one hand, a conflicted sense of self-esteem makes them strive for success. On the other hand, it causes constant doubts like “what if I haven’t achieved enough?” The real “self ” never approaches the level of the ideal “self;” it demands infinite improvement up to psychophysical exhaustion and “giving-up.”

The physiological marker of such maladaptation is a high level of cortisol in spit samples (Tarasova, 2016). The risk group consistently includes 30-35% of all children and adolescents during this time in school. As both participant and consultancy observations have shown, these pupils are prone to auto-aggressive behavior, sometimes even to the surprise of their class teacher or their parents. We called this risk group “maladaptive perfectionists.”

One can differentiate between healthy and pathological, normal and neurotic, and adaptive and maladaptive perfectionism. Whereas adaptive perfectionism is a characteristic of a harmonious personality striving for success, maladaptive perfectionism doesn’t make a person feel happy about his or her achievements. In the academic literature this idea is reflected in the terms “normal vs. neurotic,” “healthy vs. pathological,” “positive vs. negative,” “self-oriented vs. socially prescribed,” and “destructive narcissism.”

This conceptual framework has, for example, been realized in the Slaney model (2001). According to Slaney, high personality standards and striving for order comprise adaptive perfectionism. Anxiety while completing tasks, the experience of nonconformity, failing to start an activity due to the fear of bad performance, emotional instability, and the resulting difficulties with interpersonal relationships characterize maladaptive perfectionism. A maladaptive form of perfectionism is manifested in ongoing attempts to convince oneself and society of one’s own success and to gain recognition and praise from significant other people, even at the cost of exhaustion. The essence of maladaptive perfectionism lies in fundamental insatiability. A teenager assigns unreal expectations of himself or herself to significant other people and strives to live up to them to buy acceptance and approval. The teenager’s ideal “self ” never coincides with the real “self.”

The reason for such maladaptive forms of perfectionism lies in the phenomenon of conditional acceptance in a parent-child relationship (“I will love you, if you … study well, clean your room, prepare tasty food …;” “You will be good for me, if you… are well-shaped, return home on time, enter a university…”). Conditional acceptance is a variant of educational practice, a family scenario leading to maladaptive perfectionism. Maladaptive perfectionism is related to auto-aggressive manifestations, including suicide (Hamilton, 2000; Miller, 2007; Hobgood, 2011; Roxborough, 2012; Kiamanesh, 2014; Hassan, 2014; Kiamanesh, 2015; Ventriglio, 2016; Limburg, 2017; Smith, 2018; Sommerfeld, 2019; Lucas, 2019, Galvez-Sánchez, 2019; Eskander, 2020; Luca Katzenmajer-Pump, 2021).

Now let’s consider the notions of aggression and auto-aggression. We agree with S.N. Yenikolopov that aggression is a type of behavior aimed at doing physical or psychological damage or harm that goes against social standards, and brings on negative feelings, fear, and depression (Tarasova, Osnitsky, & Yenikolopov, 2016). Aggression may have an external as well as internal personality vector, and in this case, we are talking about auto-aggression (Psychology Dictionary, 1990). Hostility is a negative attitude towards other persons or a group of people that is manifested in the negative assessment of them. Both aggressiveness and hostility are factors in the propensity for aggressive behavior.

According to Freud’s classical psychoanalysis, hostility and negative feelings can be redirected inward via the mechanism of introjection. In the 1960s, there was a suggestion to differentiate between hostility directed outward and hostility directed toward oneself (self-reproach, self-effacement, and depression). At the same time, as clinical studies have shown, depressed patients are often irritable and verbally aggressive. Nasty temper and emotional lability are characteristics of depression (Lemogne, 2011). There is good reason to suggest that hostility towards others and hostility towards oneself have the same root. Both aggressiveness and hostility are factors which lead to proneness to aggressive behavior. Attempts have been made to find biological markers for hostility (Suneson, 2019). In turn, anger is an emotion that can accompany both hetero- and auto-aggression. The correlation between anger and suicidal ideas has also been shown (Hawkins, 2013).

Thus, the markers of a teenager’s maladaptive state are considered to be trait anxiety (school, interpersonal, self-esteem and/or magic), hostility, and maladaptive perfectionism. We hypothesized that in the risk group for self-esteem anxiety, the levels of aggression and hostility would positively correlate with the level of maladaptive perfectionism. The same adolescents can demonstrate auto-aggressive personality tendencies.

The purpose of the research was to find correlations between trait anxiety and aggressiveness among adolescents from different types of educational institutions. At the same time, we were interested in manifestations of maladaptive perfectionism and destructive personality tendencies as revealed by quantitative and qualitative research methods.

## Method

Our method for studying the Self-image of teens in the age group of 12-17 years old (A.M. Prikhozhan) included the following factors: 1) *Behavior*, or how the teenager’s behavior complied with adulthood’s demands (according to self-report); 2) *Intelligence and position at school*, or the self-evaluation by the teenager of his or her own intelligence and school performance, as compared to the real situation in the class; 3) *Appearance and physical attractiveness* as factors of being popular among peers (according to self-report); 4) *Anxiety*, or self-evaluation of one’s anxiety level; 5) *Communication*, or self-evaluation of communication skills; 6) *Happiness and satisfaction*, or feeling satisfied or dissatisfied with one’s life situation; 7) *Family position*, or the teenager’s satisfaction with his or her position in the family; and 8) *Self-confidence*, or the self-evaluation of one’s self-confidence.

We used the following questionnaires to measure the students’ personality traits:

- The Buss-Perry Aggression Questionnaire as adapted by S.N. Yenikolopov. The Russian-language version of this questionnaire consists of the following scales: 1) *Physical aggression*— self-report on one’s behavioral tendency for physical aggression (behavioral component); 2) *Anger*— self-report on one’s tendency for irritation (emotional component); and 3) *Hostility*— a scale consisting of two subscales, *Suspicion* and *Sensitivity to offense* (cognitive component).
- The APS-R Perfectionism Scale as adapted by S.N. Yenikolopov. The scales of the Russian-language version of the APS-R can be technically divided into those measuring healthy, adaptive perfectionism and maladaptive, neurotic perfectionism, which can be a risk factor for a number of mental disorders. *Adaptive perfectionism* leads to self-confidence, boosts self-esteem, and is reflected in concern for: *Standards*— measures one’s pursuit of high personal standards in activities and life; and *Order*— reflects tendency to maintain order, be accurate, organized. *Maladaptive perfectionism* is characterized by *Non-conformity* (the key factor according to Slaney)— the feeling of being unable to adhere to self-established high standards. The scale measures the distress caused by the discrepancy between one’s high standards and their achievement; *Relationships* — difficulties in interpersonal relationships as a consequence of too high standards and constant distress; *Procrastination/ Anxiety*, where procrastination is the propensity for delaying, the inability to start doing something, and anxiety is caused by the inability or fear of not conforming to predefined high standards. This factor reveals such personal traits as emotional instability, dependency, anxiety, and the propensity to delay things. More details about the Russian-language adaptation of AP\S-R can be found in the article by Yasnaya and Yenokolopov (2013).

This study combines quantitative survey methods with qualitative semi-projective methods. We were interested in the recurrence of the destructive personality tendencies we found in the various surveys in the results of the projective methods. Such an approach allows cross-checking the results. We used the following projective tests:

- *Incomplete sentences*. This method was used to identify the participants’ personal problems. The focus was on identifying destructive personality tendencies.
- *The Hand Test*. An interpretative projective technique used to interpret the meaning of hand poses for those surveyed. This has been used as a traditional pathopsychological technique of identifying meaningful needs, motives, and personality conflicts. The following criteria for assessment and further analysis have been identified: *Active and Passive response, Tension, Aggression, Direction, Communication, Exhibition, Dependence,* and *Crippled* (Semago, N.Ya., & Semago, M.M., 2016). Also, the test is aimed at defining *Aggression expectation* (Semago, N.Ya., & Semago, M.M., 2016).
- *Pathopsychological test*. This method was used for examination of the participants’ mental activity and their adaptation potential.
- *Extended clinical conversation*. Gathering of the teens’ psychological histories. These methods were used to examine the participants’ social situations and their adaptation potential. In addition, we addressed relatives to gather psychological histories and thus to verify manifestations of destructive personality tendencies by the participants.
- *Torrance figurative subtests*. They were used to define the level of originality of thinking. Also, the figurative subtests were analyzed in the same way as projective drawing techniques.
- *Sociometry. Demand*— the number of positive choices in the class of the given child; *Isolation*— the number of negative choices; *Status*— the difference between positive and negative choices; *Satisfaction*— the number of mutual positive choices; *Tension*— the number of mutual negative choices; *Frustration*— the number of rejected positive choices; *Egocentrism*— the number of in-demand negative choices.

The subject and homeroom teachers were surveyed about the victims of bullying at their schools. Their expert opinion was compared to the results of the participant observation.

### Methods of data analysis

We used methods of descriptive statistics for the analysis of the data. The correlation analysis (Spearman’s rank correlation coefficient) and analysis of intergroup differ-ences (Mann-Whitney U-test) were carried out.

The qualitative analysis of the meaningful needs, motives, and personality con-flicts of the participants was done based on the results of the whole range of tests.

### Participants

The research was carried out in five schools of different types: School 1 was a general secondary school in Dmitrov (comprised of so-called deviant classes); School 2 was a gymnasium in Dmitrov; *S*chool 3— a rural school in Moscow region near Dmitrov; School 4 was a school with a focus on learning English in Moscow (located at Kutu-zovsky Prospekt); and School 5 was a general secondary school in Moscow.

We defined schools 2, 4, and 5 as prestige, and schools 1 and 3 as non-prestige. The gymnasium selects children and adolescents with high aptitude, standard academic performance in main subjects, and good behavior. Children and adolescents from problematic families, and difficult teenagers whose parents or legal representatives are on the radar screen of Children’s Services, are found in the so-called deviant classes. The total number of adolescents who participated in the research was 260, including 144 boys and 116 girls. Written informed consent to participate in this study was provided by the participants’ legal guardian/next of kin. The participation was voluntary. After the psychological tests, feedback was provided upon the request of children and their parents/representatives.

### Procedure

The research consisted of two stages.

The first was a survey to define risk groups for hostility and anxiety among the adolescents. All tests except sociometry were done on an individual basis. They were planned taking into account the schedule of the school’s qualification tests, to order to avoid an increase in state anxiety among the adolescents. Sociometry was carried out in a given social group (class).

The second stage included methods of qualitative analysis of personality. The pupils from identified risk groups were paid special attention. We were interested in the recurrence of destructive personality tendencies found in the first stage in the second.

## Results

Below are the final results for the prestige and non-prestige schools (see *Tables 1* and*2*).

**Table 1:** The results of descriptive statistics on factors of propensity for aggression in prestige schools

	Factors of the Buss-Perry method		
Measures	Physical aggression	Anger	Hostility
Medium	22.98	19.33	21.80
Minimum	9.00	7.00	10.00
Maximum	41.00	31.00	40.00
Standard deviation	7.19	6.19	7.14
Median value	23.50	19.00	21.50
Mode	Multiple	Multiple	Multiple

**Table 2:** The results of descriptive statistics on factors of propensity for aggression in non-prestige schools

	**Factors of the Buss-Perry method**		
Measures	Physical aggression	Anger	Hostility
Medium	21.00	20.19	19.44
Minimum	14.00	10.00	11.00
Maximum	35.00	29.00	25.00
Standard deviation	5.34	5.04	4.44
Median value	19.50	19.50	20.50
Mode	19.00	19.00	25.00

In each school a risk group for hostility as a factor of propensity for aggressive behavior was identified. For this purpose, we divided pupils from every class into two contrasting groups according to the *Hostility* factor: one with an index of > 22 and the other with an index of < 22. The risk group accounted for 35% of the research participants in School 1, 17% in School 2, 26% in School 3, 31% in School 4, and 38% in School 5. As we can see, the number of highly hostile adolescents in School 2— the gymnasium in Dmitrov— was relatively low, while in the rest of the schools, they comprised about one third of the research participants.

Then we analyzed the differences between the highly hostile (> 22 scores) and low hostile (< 22 scores) pupils using the Mann-Whitney U-test. The most significant differences were found in School 5, the general secondary school in Moscow. Highly hostile adolescents were more worried about non-conformity with their self-imposed standards (p = 0.0006) and had more communication problems (p = 0.00007) than low hostile teenagers. The procrastination level of the highly hostile teenagers was also higher (p = 0.00004). Similar data was received in School 4 at Kutuzovsky Prospekt. Highly hostile adolescents were more worried about their non-conformity with self-imposed standards (p = 0.005) and had more communication problems (p = 0.003) than the teenagers with low hostility. This tendency was also seen in the gymnasium in Dmitrov. Highly hostile adolescents were more worried about nonconformity with their self-imposed standards (p = 0.056).

We compared highly hostile adolescents from the three prestige schools. Taking into account the Bonferroni correction, the new critical level had to be 0.05/3 = 0.17. Highly hostile pupils in the gymnasium in Dmitrov (p = 0.0013) and in the general secondary school in Moscow (p = 0.00005) had higher values of adaptive perfectionism than in the school at Kutuzovsky Prospekt. The level of procrastination in the Moscow school was higher too (p = 0.017). On the perfectionism side, both schools— those in Dmitrov and in Moscow— didn’t differ from each other.

In these three schools some statistically important correlations between the Buss-Perry method and APS-R Perfectionism Scale were identified (see *Tables 3, 4* and *5*).

**Table 3:** The results of the correlation analysis of the scale values from the aggression questionnaire and the perfectionism scale in the gymnasium in Dmitrov

Scales	Spearman’s rank correlation coefficient		
	Physical aggression	Anger	Hostility
	r	r	r
Non-conformity	.31*	.49***	.63***
Relationships	n.s.	.41**	.55***
Procrastination / Anxiety	n.s.	.51***	n.s.

Symbols: n.s.— a non-significant value; symbols indicate the significance level: *— p < 0.05; **— p < 0.01; ***— p < 0.001.

**Table 4:** The results of the correlation analysis of the scale values from the aggression questionnaire and the perfectionism scale in the school at Kutuzovsky Prospekt

Scales	Spearman’s rank correlation coefficient		
	Physical aggression	Anger	Hostility
	r	r	r
Non-conformity	n.s.	.39**	.44***
Relationships	n.s.	n.s.	.43***
Procrastination /Anxiety	n.s.	n.s.	n.s.

**Table 5:** The results of the correlation analysis of the scales values from the aggression questionnaire and the perfectionism scale in the general secondary school in Moscow

Scales	Spearman’s rank correlation coefficient		
	Physical aggression	Anger	Hostility
	r	r	r
Non-conformity	.28*	.31**	.46***
Relationships	n.s.	.36**	.51***
Procrastination / Anxiety	.27*	.28*	.52***

We can see slight differences between the schools, but in general the picture is similar. The school at Kutuzovsky Prospekt has a long tradition of focusing on success. The gymnasium in Dmitrov also has a long history with its own cherished traditions which, however, may be more associated with the achievements of their pupils. Since 2007 the gymnasium in Dmitrov has been in the top 10 schools of the Moscow region by annual results. A similar picture is seen at School 5.

How are the results on adaptive and maladaptive perfectionism correlated with the components of Self-concept? The correlations appear in *Table 6*.

**Table 6:** The results of the correlation analysis of the Self-concept components and maladaptive perfectionism in prestige schools

Schools	Spearman’s rank correlation coefficient		
	Non-conformity and self-assessment of the school situation	Non-conformity and self-assessment of appearance	Non-conformity and self-assessment of communication skills
	r	r	r
School 2	–.45***	–.26*	n.s.
School 4	–.48***	–.29*	–.44***
School 5	–.42***	n.s.	–.39**

However, the strongest correlations between the components of Self-concept and perfectionism were found in School 2, *i.e*., in the gymnasium in Dmitrov (see *Table 7*).

**Table 7:** The results of the correlation analysis of the Self-concept components and maladaptive perfectionism in the gymnasium in Dmitrov

Scales	Spearman’s rank correlation coefficient		
	Order (adaptive perfectionism scale)	Interpersonal relationships	Procrastination/ Anxiety
	r	r	r
Behavior	.64**	–.62*	–.70*
Intelligence	.54*	–.72*	–.74***
Situation at school	.62**	–.74***	–76***
Appearance	.53*	n.s.	n.s.
Anxiety	–.70**	.73***	.76***
Communication	.66**	–.53*	–.69*
Happiness and satisfaction	.61**	–.58*	–.62*
Family situation	n.s.	–.59*	n.s.
Self-confidence	.66**	n.s.	–.48*

As these results show, almost all the components of Self-concept were crucial for self-esteem anxiety among the gymnasium students.

We then decided to look at the percentages within the trait anxiety risk group. We calculated the number of teenagers from the risk group expressing the *Anxiety, self-assessment of the anxiety level*, factor from the Self-concept research method for the 12-17 age group by A.M. Prikhozhan. For this purpose, we divided the pupils from every class who expressed the *Anxiety, self-assessment of the anxiety level* factor into two contrasting groups: one with scores of > 8 and the other with scores of < 8 (Prikhozhan, 2009. Appendix 10). The study showed that there were no pupils in the gymnasium who had both a high level of emotional well-being and a low level of adaptive anxiety. In the other schools the number of teenagers from the risk group varied from 16% to 20%.

Some interesting gender differences were identified among the gymnasium students. Girls were more anxious than boys as measured by the Procrastination/Anxiety scale (p = 0.01). Girls also had higher levels of anger (p = 0.02). This can be explained by the fact that, starting from the 5^th^ grade, when pupils are distributed according to their specialization, classes come onto the school administration’s radar screen. Participant observation and conversations with teachers showed that the levels of anxiety among teachers at this school are also very high. The teachers have a strong focus on performance and thus give a lot of tests. In this context, the Spielberger trait anxiety questionnaire was added to those given at the gymnasium in Dmitrov. Although we tried to take into account the school test schedule in order to exclude the increase of situational anxiety as much as possible, most of the research participants showed the high level of anxiety (72%). Also, none of the gymnasium classes which participated in the research contained pupils with the low level of anxiety. Girls were much more anxious than boys, according to the Spielberger test (p = 0.002).

Consider this case from the gymnasium: a 14-year old girl with the highest scores on the non-conformity scale on the perfectionism questionnaire. This score means she doesn’t measure up to her own high personality standards. Her difficulties with interpersonal relationships as a result of too high standards are also high. According to her scores on the *Anxiety* scale of the Prikhozhan questionnaire, this girl falls into the risk group (borderline state, *i.e.,* neurotization level; negative level of self-attitude).

In addition, her non-conformity to her own standards is seen in her incomplete sentences test. Some examples: “It’s better for me to study with people of the same knowledge level as mine;” “The teachers at school underestimate my skills;” “People I study with are too intelligent;” “I think my dad rarely can be attentive;” “I would have been very happy, if I had been more myself when communicating with others;” “In comparison to most of other families my family is too religious;” and “When I was a child, I believed in God too much.” Her hand test results also revealed high values for the *Anxiety* and *Aggressiveness* criteria, and a high level of expectation of aggressive behavior was determined. It should be added that if we analyze her Torrance figura-tive subtest as a projective drawing technique, we can see clear hatching and blackening. This is an indirect characteristic of a high level of anxiety, or neurotization.

We did not administer the perfectionism survey at the rural school and in the deviant classes because the pupils found the questions too complicated. Correlations for other psychological characteristics were found, however. In the general secondary school (School 1) the levels of physical aggression negatively correlated with self-assessment of one’s behavior as normal (r = –0.54, p < 0.05). Hostile teenagers more often assessed their own intelligence (r = –0.58, p < 0.05) and communication skills (r = –0.77, p < 0.001) at a low level. In this case the assessment reflected the reality: according to the expert opinion of the teachers and sociometry results, hostile pupils are isolated in their social group (class) (r = 0.52, p < 0.05).

Similar results were gotten at the rural school (School 3). Hostile teenagers were more often not self-confident (r = –0.63, p < 0.05). The pupils who lacked self-confi- dence assessed their intelligence at a low level (r = 0.68, p < 0.05). As in the general secondary school in Dmitrov, in this case the assessment reflected reality: hostile pupils are isolated within the social group (class) (r = 0.69, p < 0.05). Therefore, hostile adolescents in the rural and the so-called “deviant” schools more often had self-esteem problems and were isolated in their social group (class). The crucial factors were intelligence, appearance, family situation, and happiness.

Previously we identified the pupils who fall into hostility risk groups by using survey methods. Now, we found that the same teenagers demonstrated destructive tendencies in the pathopsychological test, the hand test, the Torrance figurative subtest, and the participant observation data. Therefore, the qualitative analysis methods confirmed the survey methods.

Take the case of F.M., a boy from the 8^th^ grade at the rural school, who is in the risk group for hostility and aggressiveness (having scored the highest values on the Buss-Perry Questionnaire). This teenager breaks all rules and was the object of constant complaints from his teachers and classmates. He heated his house key with a lighter and scorched classmates with it. The teachers also noted that F.M. often brought sharp and cutting things to the school and walked around with a razor blade in his mouth.

His social situation was as follows: F.M. lives with his mother, her father-in-law, and a younger sister. He doesn’t have contact with his biological father. According to the teachers, F.M. suffered a family tragedy two years ago. The boy and his younger brother decided to play snowballs at home. They crumpled paper imitating snowballs. The boys were overwhelmed by the game when one of the “snowballs” fell behind the microwave. There was an electrical short and the paper went up in flames. The younger brother rushed up the stairs to call for the mother, who was upstairs with the boys’ younger sister. She ordered the two younger children to stay in the room and went down to fight the fire. She didn’t succeed, and the house got engulfed by flames. Since she was pregnant, she realized that she needed to get out of the house immediately, pushed out F.M., and got out herself. The two younger children died in the fire. After the accident she blamed F.M. for a long time.

F.M.’s hand test results showed that aggressive and dominant tendencies in F.M. dominate his social cooperation mindset. This also means a high level of hostility. Here are the examples of his answers in the hand test: “The man is killed, like that, into the Adam’s apple;” “A torch and a man in the dark, it’s raining. The buddy got out of the house and followed the victim. He is a maniac; he will kill the first one whom he meets. But not a child— a man or a woman, no difference;” “Somebody is sleeping or dead. She has cut her veins, a suicider. Somebody offended her, she came home, took a knife, cut her veins, and died;” “A kid goes up the crane. He is completing a task from the Blue Whale challenge.”

The death theme was also present in his incomplete sentences test. For example: “I would do everything to forget the fire;” “I would have been much happier if not for the fire;” “My dad rarely was at home;” “Hope for death;” “If everybody is against me, I hit them.” During the conversation he admitted to playing “kill yourself ” and the Blue Whale challenge. The boy said that he had completed tasks from these games: he ran between two heavy-duty trucks and climbed up the crane to receive money for that.

It should be noted that analysis of the Torrance figurative subtest can be used as a projective test. Analyzing the Torrance figurative subtests as projective techniques may reveal destructive personality tendencies.

As shown above, there is not much difference between the pictures of anxiety and aggressiveness in the rural and so-called deviant schools. Also, no statistically meaningful difference was found between urban and rural schools for aggression and self-esteem anxiety indicators. However, in the rural school the positive assessment of the school situation by the pupils tended to be higher (p = 0.05). This may be related to the rural area being less developed in terms of entertainment and technology, so-called digitalization. For adolescents from a small village, a school is one of the important places where they can communicate with friends, take part in different events, and “have a rest from home and parents.” The educational process doesn’t imply the use of computers and the Internet. Therefore, the school is perceived by pupils not only as a place where they get knowledge, but also as a social circle.

At the same time, pupils from the urban schools are exposed to higher requirements in terms of technical support of the educational process. New school technologies require pupils to have Internet access, and are used to monitor tasks, and transmit homework, news, and test scores in electronic form. This results in less face-to-face communication. Teachers are unable to pay sufficient time and attention to the adolescents because of the avalanche of paperwork. That’s why pupils from the urban school don’t see the school situation as satisfactory; they don’t get adequate attention and communication at school, which is so much needed at their age. According to the participant observation, a mobile phone is used more actively in an urban school than in a rural school. We can probably talk about the side effects of digitalization for the psychological and personality health of adolescents.

According to the results of the sociometry in the prestige schools (schools 2, 4 and 5), the Buss-Perry results were correlated with the sociometric index *Isolation* (from r = 0.24 to r = 0.27 if p < 0.05).

## Discussion

To sum up, in both the prestige and non-prestige schools alike, the more hostile teenagers were isolated in their social group (class) to a greater or lesser degree. In addition, they had problems with self-esteem and in forming their Self-concept in general, as well as pronounced destructive personality tendencies in some cases. However, the pictures of a pupil’s self-esteem from a prestige school and a non-prestige school were different. The teenagers from the hostility risk group in the non-prestige schools assessed their intelligence and communication skills at a low level. In this case, according to the teachers’ expert opinion and the participant observation data, the assessment corresponded to reality. In the prestige educational institutions, we observed a different picture of self-esteem anxiety. Objectively, the pupils’ achievements were great, and even brilliant, but the teenagers constantly procrastinated and didn’t believe in success, which led to communication problems with peers and adults.

The maladaptive perfectionism phenomenon has its roots both in the family and in society in general. By teen age, standards and settings established by the family will have been interiorized. Normally, adaptive perfectionism should have been formed, namely, a tendency to be accurate and organized, take adequate responsibility, and have a harmonious self-image. Teenagers from neurotic families develop maladaptive perfectionism when they feel that they don’t live up to their own standards. Their inability to meet self-established high standards leads to and maintains a high anxiety level.

We shouldn’t forget the cult of success in modern society, including in relation to appearance. Non-conformity to an “ideal” image may be the reason for bullying. The media and Internet play critical roles in this, since their influence combines with the already aggravated teenage crisis. Teenagers are not free of maximalist conceptual mindsets and are more conformable than adults. The imposition of standards, such as the ideal body standards, leads to eating disorders. In this case, self-esteem anxiety and maladaptive perfectionism become intermediating factors for the pupils’ maladaptive behavior.

The imposition of unrealistic standards may also take place in the real world, but on the Internet the tendency is less controlled by parents, even if they have a positive emotional connection with their children. In addition, there is power in the printed word. The Internet makes it possible to form and establish a social identity based on personality traits that clinical psychologists usually characterize as pathological. A wide diversity of online communities opens the door for overindulgence in self-expression by socially marginal and clinically pathological personalities. In turn, pathological posts act as magnets for attention. Teenagers, as is well known, have idols, and these are usually not their parents. Therefore, it is very important that idols like Billie Eilish show a psychologically sound and harmonious self-image.

We can thus identify some negative side effects of digitalization for the psychological and personality health of adolescents. First, there is the Blue Whale challenge. The Internet is the place where young people encounter stories and talks about suicide, and such influence, especially in the form of discussion clubs, fosters suicidal thoughts. Second, adolescents can strive for digital distraction from anxiety and distress arising from their live communication with peers. Regulation of emotions is an important skill that develops in childhood and adolescence, when people learn how to deal with strong emotions by recognizing them and developing inner regulatory processes. Psychological theory has widely admitted that regulation of emotions is an important component of mental health, while any problems with it lead to different psychopathological disorders, including depression. If a person escapes from live communication to living on the Internet, he or she will never learn how to communicate in real space.

Time spent online displaces face-to-face communication. A vicious circle forms. For these people such displacement of interpersonal communication with network communication, carried out in order to escape threatening situations, can be cyclically reinforced, and this makes a person even more isolated and anxious. Thirdly, there is the anxiety arising as a result of fear of not being “online.” Social media (text and instant messages, email) have become the main methods of communication for most adolescents, and interruption of such networks can heighten the level of anxiety. When a teenager doesn’t receive a response to his or her message, he or she can feel the fear of being rejected.

Seven pupils from the identified risk group for hostility were the most isolated in the class. According to the results of participant observation, their psychological histories, and the teachers’ expert opinions, these same seven pupils were the victims of bullying and cyberbullying. In this study we didn’t focus on bullying and cyberbul-lying, but other research we have carried out has shown that victims of bullying have similar characteristics: they are stably and highly hostile and highly anxious, and have problems with self-esteem (2016). There is danger of their victimization as well as of the victim turning into an aggressor in the future.

This result corresponds to the results of other studies. Researchers have shown a correlation between maladaptive perfectionism and suicide risk among children and adolescents, with the intermediating factors being bullying and social despair (Roxborough, 2012).

Cyberbullying, like conventional bullying, rapidly becomes part of our life. (Ju-vonen, 2008; Kowalski, 2014; John, 2018). Previous and current experiences of traditional humiliation are associated with both the victims and performers of cyberbul-lying.

Cyberbullying can be worse than bullying. According to some data, the prevalence rate of cyberbullying ranges from 6% to 35-40% (Aboujaoude, 2015). In one study, where participants were picked from a popular website, the prevalence rate was significantly higher— 72% (Juvonen, 2008). Bullying on the Internet usually takes place in late teenage, starting from 14 years old, when pupils spend more time on their smartphones and social media. It is reinforced by Internet usage for more than three hours per day, the use of messenging, web-cameras, posting personal information, etc. Cyberbullying can reach more people than traditional acts of humiliation because it takes place in cyberspace, which has weakened or no social control. Those guilty of cyberbullying also have a degree of anonymity that is impossible in traditional acts of humiliation, and the potential suffering and embarrassment of a victim is more pronounced. One can bully a classmate in his or her own house or in any other place at any time, and even if he or she deletes the profile, messages are often accumulated and read by a wide audience. Besides, it is not easy to just delete information from the web.

Cyberbullying is a proven predictor of suicidal behavior among adolescents. Victims of cyberbullying and humiliation at school have higher risks of suicidal thoughts, plans, and attempts. For example, in the Zaborskis survey, 39% of adolescents reported that over the past year they had been experiencing emotions that hindered their ordinary course of activities; 18% of adolescents had considered a suicide attempt; 12% of adolescents had drawn up a suicide plan; and 9.5% of adolescents had attempted a suicide (Zaborskis, 2019).Victims of humiliation often have mental health problems, shown by depression symptoms, self-injury, and suicidal behavior (Krešic Coric, 2020). Thus, cyberbullying poses a serious risk to pupils’ mental health.

Even in the face of these results, it is important to admit that the social character of the Internet also makes it an effective resource for psychotherapeutic support. Active participation in online support groups can potentially result in lower stress because it provides wider access to helpful messages. The nature of the influence of the Internet on behavior depends on the particular information to which access is provided and on the people with whom one is in contact. To sum up, there is an ongoing process of adaptation to these new technologies, and, thus, the side effects of civilization that bring both the positive and negative with it.

Whatever the reason for teenage anxiety and maladaptation, professional psychological assistance is highly recommended. For example, schools have to engage clinical psychology experts to solve such problems.

## Conclusion

1. Our empirical study has shown that the markers of a pupil’s maladaptive state are trait anxiety (school, interpersonal, self-esteem), hostility, and maladaptive perfectionism.
2. Schools with a selection principle of admission have been traditionally called prestige schools. In prestige and non-prestige schools alike, hostile teenagers were more often isolated in their social group (class) to a greater or lesser degree. They had problems with self-esteem and in forming their Self-concept in general, as well as pronounced destructive personality tendencies in some cases. However, the pictures of a pupil’s trait anxiety from a prestige school and a non-prestige school were different. Hostile teenagers from non-prestige schools more often assessed their intelligence and communication skills at a low level. In this case, according to the teachers’ expert opinion and the participant observation data, that assessment corresponded to reality.
3. In the prestige educational institutions, we observed a different picture of trait anxiety. Objectively, the pupils’ achievements were great, but the teenagers constantly procrastinated, didn’t believe in success, and had communication problems.
4. About one third of the research participants (from 26% to 38% in different classes) from four out of the five schools fell into the hostility risk group. According to the results of the pathopsychological test, the hand test, the Torrance figurative test, and the participant observation data, the same teenagers demonstrated pronounced destructive tendencies.

## Limitations

This research has been restricted by a relatively small number of participants. We tried to offset this by carrying out pathopsychological tests with the research participants from the risk groups for hostility and aggressiveness.
